# Robust Variable Selection and Estimation Based on Kernel Modal Regression

**DOI:** 10.3390/e21040403

**Published:** 2019-04-16

**Authors:** Changying Guo, Biqin Song, Yingjie Wang, Hong Chen, Huijuan Xiong

**Affiliations:** College of Science, Huazhong Agricultural University, Wuhan 430070, China

**Keywords:** modal regression, maximum correntropy criterion, variable selection, reproducing kernel Hilbert space, generalization error, 62J02, 68T05, 62F35

## Abstract

Model-free variable selection has attracted increasing interest recently due to its flexibility in algorithmic design and outstanding performance in real-world applications. However, most of the existing statistical methods are formulated under the mean square error (MSE) criterion, and susceptible to non-Gaussian noise and outliers. As the MSE criterion requires the data to satisfy Gaussian noise condition, it potentially hampers the effectiveness of model-free methods in complex circumstances. To circumvent this issue, we present a new model-free variable selection algorithm by integrating kernel modal regression and gradient-based variable identification together. The derived modal regression estimator is related closely to information theoretic learning under the maximum correntropy criterion, and assures algorithmic robustness to complex noise by replacing learning of the conditional mean with the conditional mode. The gradient information of estimator offers a model-free metric to screen the key variables. In theory, we investigate the theoretical foundations of our new model on generalization-bound and variable selection consistency. In applications, the effectiveness of the proposed method is verified by data experiments.

## 1. Introduction

Variable selection has attracted increasing attention in the machine learning community due to the massive requirements of high-dimensional data mining. Under different motivations, many variable selection methods have been constructed and shown promising performance in various applications. From the viewpoint of hypothesis function space, there are mainly two types of variable selection approaches with respect to linear assumption and nonlinear additive assumption, respectively. For the linear model assumption, variable selection algorithms are usually formulated based on the least-squares empirical risk and the sparsity-induced regularization, which include Least Absolute Shrinkage and Selection Operator (Lasso) [1], Group Lasso [2] and Elastic net [3] as special examples. For the nonlinear additive model assumption, various additive models have been developed to relax the linear restriction on regression function [4,5]. It is well known that additive models enjoy the flexibility and interpretability of their representation and can remedy the curse of dimensionality of high-dimensional nonparametric regression [6,7,8]. Typical examples of additive models include Sparse Additive Models (SpAM) [9], Component Selection and Smoothing Operator (COSSO) [10] and Group Sparse Additive Models (GroupSpAM) [11]. Most of the above approaches are formulated under Tikhonov regularization scheme with special hypothesis function space (e.g., linear function space, nonlinear function space with additive structure).

More recently, some works have been made in [12,13,14,15] to alleviate the restriction on the hypothesis function space, which just require that the regression function belongs to a reproducing kernel Hilbert space (RKHS). In contrast to the traditional structure assumption on regression function, these methods identify the important variable via the gradient of kernel-based estimator. There are two strategies to improve the model flexibility through the gradient information of predictor. One follows the learning gradient methods in [13,14,16], where the functional gradient is used to construct the loss function for forming the empirical risk. Under this strategy, two model-free variable selection methods are presented by combining the error metric associated with the gradient information of estimator and the coefficient-based $\ell_{2,1}$-regularizer in [13] and ${∥\cdot ∥}_{K}$-regularizer in [14], respectively. In particular, the variable selection consistency is also established based on the properties of RKHS and mild parameter conditions (e.g., the regularization parameter, the width of kernel). The other follows the structural sparsity issue in [15,17], where the functional gradient is employed to construct the sparsity-induced regularization term. Rosasco et al. in [17] proposes a least-squares regularization scheme with nonparametric sparsity, which can be solved by an iterative procedure associated with the theory of RKHS and proximal methods. Magda et al. [15] introduces a nonparametric structured sparsity by considering two regularizers based on partial derivatives and offers its optimization with the alternating direction method of multiples (ADMM) [18]. Moreover, to further improve the computation feasibility, a three-step variable selection algorithm is developed in [12] with the help of the three building blocks: kernel ridge regression, functional gradient in RKHS, and a hard threshold. Meanwhile, the effectiveness of the proposed algorithm in [12] is supported by theoretical guarantees on variable selection consistency and empirical verification on simulated data.

Despite the aforementioned methods showing promising performance for identifying the active variables, all of them rely heavily on the least-squares loss under the MSE criterion, which is sensitive to non-Gaussian noise [19,20], e.g., the heavy-tailed noise, the skewed noise, and outliers. In essence, learning methods under MSE aim to find an approximator to the conditional mean based on empirical observations. When the data are contaminated by a complex noise without zero mean, the mean-based estimator is difficult to reveal with the intrinsic regression function. This motivates us to formulate a new variable selection strategy in terms of other criterion with respect to different statistical metric (e.g., the conditional mode). Following the research line in [12,19], we consider a new robust variable selection method by integrating the issues of modal regression (for estimating the conditional mode function) and variable screening based on functional derivatives. To the best of our knowledge, this is the first paper to address robust model-free variable selection.

Statistical models for learning the conditional mode can be traced back to [21,22], which include the local modal regression in [23,24] and the global modal regression in [25,26,27]. Recently, the idea of modal regression has been successfully incorporated into machine learning methods from theoretical analysis [19] and application-oriented studies (e.g., cognitive impairment prediction [20] and cluster estimation [28]). Particularly, Feng et al. [19] considers a learning theory approach to modal regression and illustrates some relations between modal regression and learning under the maximum correntropy criterion [29,30,31]. In addition, Wang et al. [20] formulates a regularized modal regression (RMR) under modal regression criterion (MRC), and establishes its theoretical characteristics on generalization ability, robustness, and sparsity. It is natural to extend the RMR under linear regression assumption to general model-free variable selection setting.

Inspired by recent works in [12,19], we propose a new robust gradient-based variable selection method (RGVS) by integrating the RMR in RKHS and the model-free strategy for variable screening. Here, the kernel-based RMR is used to construct the robust estimator, which can reveal the truly conditional mode, even when facing data with non-Gaussian noise and outliers. Moreover, we evaluate the information quantity of each input variable by computing the corresponding gradient of estimator. Finally, a hard threshold is used to identify the truly active variables after offering the empirical norm of each gradient associated with hypothesis function. The above three steps assure the robustness and flexibility of our new approach.

To better highlight the novelty of RGVS, we present Table 1 to illustrate its relation with other related methods, e.g., linear models (Lasso [1], RMR [20]), additive models (SpAM [9], COSSO [10]), and General variable selection Method (GM) [12].

Our main contributions can be summarized as follows.

- We formulate a new RGVS method by integrating the RMR in RKHS and the model-free strategy for variable screening. This algorithm can be implemented via the half-quadratic optimization [32]. To our knowledge, this algorithm is the first one for robust model-free variable selection.
- In theory, the proposed method enjoys statistical consistency on regression estimator under much general conditions on data noise and hypothesis space. In particular, the learning rate with polynomial decay $O(n^{-\frac{2}{5}})$ is obtained, which is faster than $O(n^{-\frac{1}{7}})$ in [20] for linear RMR. It should be noted that our work is established under the MRC, while all previous model-free methods are formulated under the MSE criterion. In addition, variable selection consistency is obtained for our approach under a self-calibration condition.
- In application, the proposed RGVS shows the empirical effectiveness on both simulated and real-world data sets. In particular, our approach can achieve much better performance than the model-free algorithm in [12] for complex noise data, e.g., containing Chi-square noise, Exponential noise, and Student noise. Experimental results together with theoretical analysis support the effectiveness of our approach.

The rest of this paper is organized as follows. After recalling the preliminaries of modal regression, we formulate the RGVS algorithm in Section 2. Then, theoretical analysis, optimization algorithm, and empirical evaluation are provided from Section 3 to Section 5 respectively. Finally, we conclude this paper in Section 6.

## 2. Gradient-Based Variable Selection in Modal Regression

Let $X\in R^{p}$ and Y∈R be a compact input space and an output space, respectively. We consider the following data-generating setting

(1) $$y=f^{*}\left(x\right)+\epsilon ,$$

where x∈X, y∈Y and ϵ is a random noise. For the feasibility of theoretical analysis, we denote the intrinsic distribution of (x,y)∈Z:=(X,Y) generated in (1) as ρ. Let $z={\left\{\left(x_{i},y_{i}\right)\right\}}_{i=1}^{n}\in Z^{n}$ be empirical observations drawn independently according to the unknown distribution ρ. Unlike sparse methods with certain model assumption (e.g., Lasso [1], SpAM [9]), the gradient-based sparse algorithms [12,13] mainly aim at screening out the informative variables according to the gradient information of intrinsic function. For input vector $u={\left(u_{1},\ldots ,u_{p}\right)}^{T}\in R^{p}$, the variable information is characterized by the gradient function $g_{j}^{*}\left(u\right):=\partial f^{*}\left(u\right)/\partial u_{j}$. Clearly, $g_{j}^{*}\left(u\right)=0$ implies that the *j*-th variable is uninformative [12,17]. Considering an $\ell_{2}$-norm measure on the partial derivatives, we denote the true active set as

(2) $$\begin{array}{c} S^{*}=\{j:∥g_{j}^{*}{∥}_{2}^{2}>0\}, \end{array}$$

where $∥g_{j}^{*}{∥}_{2}^{2}=\int_{X}{\left(g_{j}^{*}\left(x\right)\right)}^{2}d\rho_{X}\left(x\right)$ and $\rho_{X}$ is the marginal distribution of ρ.

Indeed, all the gradient-based variable selection algorithms [12,13,17] are constructed under Tikhonov regularization scheme in RKHS $H_{K}$ [33,34]. The RKHS $H_{K}$ associated with the Mercer kernel *K* is the closure of the linear span of $\left\{K_{x}:=K\left(x,\cdot \right):x\in X\right\}$. Such a Mercer kernel K:X×X→R is a symmetric and positive semi-definite function. Denote $<\cdot ,\cdot {>}_{K}$ as the inner product in $H_{K}$, the reproducing properties of RKHS means $<f,K_{x}{>}_{K}=f\left(x\right),\forall f\in H_{K}$.

### 2.1. Gradient-Based Variable Selection Based on Kernel Least-Squares Regression

In this subsection, we recall the gradient-based variable selection algorithm in [12] associated with least-squares error metric. When the noise ϵ in (1) satisfies E(ϵ|X)=0 (i.e., Gaussian noise), the regression function equals to the conditional mean, which can be represented by

(3) $$\begin{array}{c} f^{*}\left(x\right)=E\left(Y|X=x\right)=\int_{Y}yd\rho_{Y|X=x}\left(y\right). \end{array}$$

Here $\rho_{Y|X=x}$ denotes the conditional distribution of *Y* given *x*. Theoretically, the regression function $f^{*}$ in (3) is the minimizer of expected least-squares risk

$$E\left(f\right)=\int_{Z}{\left(y-f\left(x\right)\right)}^{2}d\rho \left(x,y\right).$$

As ρ is unknown in practice, we cannot get $f^{*}$ directly by minimizing E(f) over certain hypothesis space. Given training samples z, the empirical risk with respect to the expected risk E(f) is denoted by

(4) $$E_{z}\left(f\right)=\frac{1}{n}\sum_{i=1}^{n}{\left(y_{i}-f\left(x_{i}\right)\right)}^{2}.$$

The gradient-based variable selection algorithm in [12] depends on the estimator defined as below:

(5) $$\begin{array}{c} \tilde{f_{z}}=\arg\min_{f\in H_{K}}\{E_{z}\left(f\right)+{\lambda ||f||}_{K}^{2}\}, \end{array}$$

where λ>0 is the regularization parameter and ${\left||f|\right|}_{K}$ is the kernel-norm of *f*. The properties of RKHS [33] assure that

$$\tilde{f_{z}}\left(x\right)=\sum_{i=1}^{n}{\tilde{\alpha }}_{i}K\left(x_{i},x\right)={\left({\tilde{\alpha }}_{z}\right)}^{T}K_{n}\left(x\right),$$

where $K_{n}\left(x\right)={\left(K\left(x_{1},x\right),\cdots ,K\left(x_{n},x\right)\right)}^{T}\in R^{n}$ and ${\tilde{\alpha }}_{z}={\left({\tilde{\alpha }}_{1},\cdots ,{\tilde{\alpha }}_{n}\right)}^{T}\in R^{n}$. Denote $K=\left(K_{n}\left(x_{1}\right),\cdots ,K_{n}\left(x_{n}\right)\right)\in R^{n\times n}$ and $Y={\left(y_{1},\ldots ,y_{n}\right)}^{T}\in R^{n}$, the closed-form solution is

$$\begin{array}{c} {\tilde{\alpha }}_{z}={\left(K^{T}K+n\lambda K\right)}^{-1}KY. \end{array}$$

Following Lemma 1 in [12], for any j∈{1,⋯,p}, we have ${\tilde{g}}_{j}\left(x\right)={\left({\tilde{\alpha }}_{z}\right)}^{T}\partial_{j}K_{n}\left(x\right)$, where

$$\partial_{j}K_{n}\left(u\right)={\left(\frac{\partial K(x_{1},u)}{\partial u_{j}},\ldots ,\frac{\partial K(x_{n},u)}{\partial u_{j}}\right)}^{T},u={\left(u_{1},\ldots ,u_{p}\right)}^{T}\in X.$$

After imposing the empirical norm on ${\tilde{g}}_{j}$, i.e.,

$$∥{\tilde{g}}_{j}{∥}_{n}^{2}=\frac{1}{n}\sum_{i=1}^{n}{\left({\tilde{g}}_{j}\left(x_{i}\right)\right)}^{2},$$

we get the estimated active set

$$\tilde{S}=\{j:∥{\tilde{g}}_{j}{∥}_{n}^{2}>v_{n}\},$$

where $v_{n}$ is a pre-configured constant for variable selection.

The general variable selection method has shown some theoretical advantages in [12], e.g., the representation flexibility and the computation feasibility. However, the gradient-based method [12] may result in a degraded performance for real-world data without the zero-mean noise condition. Inspired by the modal regression [19,35] to learn the conditional mode, we propose a new robust gradient-based variable selection method under much general noise condition.

### 2.2. Robust Gradient-Based Variable Selection Based on Kernel Modal Regression

Unlike the traditional zero-mean noise assumption [12,17], the modal regression requires that the conditional mode of random noise ϵ is zero for any x∈X, i.e.,

$$\mathrm{mode}\left(\epsilon |X=x\right)=\arg\max_{t\in R}P_{\epsilon |X}\left(t|X=x\right)=0,$$

where $P_{\epsilon |X}$ is the conditional density of ϵ conditioned on *X*. In fact, this assumption imposes no restrictions on conditional mean, and can include the heavy-tailed noise, the skewed noise, and outliers. Then, we can verify that the mode-regression function

(6) $$f^{*}\left(x\right)=\mathrm{mode}\left(Y|X\right)=\arg\max_{t\in R}P_{Y|X}\left(t|X=x\right),$$

where $P_{Y|X}\left(\cdot |X\right)$ denotes the conditional density of *Y* conditioned on x∈X. It is worth noting that $P_{Y|X}\left(\cdot |X=x\right)$ is assumed to be unique and existing here. As shown in [19,20], $f^{*}$ in (6) is the maximizer of the MRC over all measurable functions, which is defined as

$$R\left(f\right)=\int_{X}P_{Y|X}\left(f\left(x\right)|X=x\right)d\rho_{X}\left(x\right).$$

The maximizer of R(f) is difficult to be obtained since both $P_{Y|X}$ and $\rho_{X}$ are unknown. Fortunately, Theorem 5 of [19] has proved that $R\left(f\right)=P_{E_{f}}\left(0\right)$, where $P_{E_{f}}\left(0\right)$ is the density function of $E_{f}=Y-f\left(x\right)$ at 0 and which can be easily approximated by the kernel density method [20]. With the help of modal kernel $K_{\sigma }:R\times R\to R$ for the density estimation, we can formulate an empirical kernel density estimator ${\hat{P}}_{E_{f}}$ at 0

(7) $$\begin{array}{ccc} {\hat{P}}_{E_{f}}\left(0\right) & = & \frac{1}{n\sigma }\sum_{i=1}^{n}K_{\sigma }\left(y_{i}-f\left(x_{i}\right),0\right)=\frac{1}{n\sigma }\sum_{i=1}^{n}K_{\sigma }\left(y_{i},f\left(x_{i}\right)\right):=R_{z}^{\sigma }\left(f\right). \end{array}$$

Setting $\phi \left(\frac{y-f(x)}{\sigma }\right):=K_{\sigma }\left(y,f\left(x\right)\right)$, we get the corresponding expected version

(8) $$R^{\sigma }\left(f\right)=\frac{1}{\sigma }\int_{X\times Y}\phi \left(\frac{y-f(x)}{\sigma }\right)d\rho \left(x,y\right).$$

In addition, the modal regression also can be interpreted by minimizing a mode-induced error metric [19]. When ϕ(u)≤ϕ(0) for any u∈R, the mode-induced loss can be defined as

$$L_{\sigma }\left(y-f\left(x\right)\right)=\sigma^{-1}\left(\phi \left(0\right)-\phi \left(\left(y-f\left(x\right)\right)/\sigma \right)\right),$$

which is related closely with the correntropy-induced loss in [19,36]. Given training samples $z={\left\{\left(x_{i},y_{i}\right)\right\}}_{i=1}^{n}$, we can formulate the RMR in RKHS as

(9) $$f_{z}=\arg\max_{f\in H_{K}}\left\{R_{z}^{\sigma }\left(f\right)-{\lambda ||f||}_{K}^{2}\right\},$$

where λ>0 is a turning parameter that controls the complexity of the hypothesis space, and ${∥f∥}_{K}^{2}={\langle f,f\rangle }_{K}$ is the kernel-norm of $f\in H_{K}$.

Denote $\hat{\alpha }={\left({\hat{\alpha }}_{1},\ldots ,{\hat{\alpha }}_{n}\right)}^{T}\in R^{n}$, $K_{n}\left(x\right)={\left(K\left(x_{1},x\right),\ldots ,K\left(x_{n},x\right)\right)}^{T}\in R^{n}$ and $K={\left(K\left(x_{i},x_{j}\right)\right)}_{i,j=1}^{n}\in R^{n\times n}$. From the representer theorem of kernel methods, we can deduce that

$$f_{z}\left(x\right)=\sum_{i=1}^{n}{\hat{\alpha }}_{z,i}K\left(x_{i},x\right)={\hat{\alpha }}_{z}^{T}K_{n}\left(x\right),$$

with

(10) $${\hat{\alpha }}_{z}=\arg\max_{\alpha \in R^{n}}\left\{\frac{1}{n\sigma }\sum_{i=1}^{n}\phi \left(\frac{y_{i}-K_{n}^{T}\left(x_{i}\right)\alpha }{\sigma }\right)-\lambda \alpha^{T}K\alpha \right\}.$$

From Lemma 1 in [12], we know that for any $f\in H_{K}$ and $u={\left(u_{1},\ldots ,u_{p}\right)}^{T}\in R^{p}$,

$${\hat{g}}_{j}\left(u\right)=\frac{\partial f(u)}{\partial u_{j}}={\langle f,\frac{\partial K(u,\cdot )}{\partial u_{j}}\rangle }_{K}.$$

The empirical measure on gradient function ${\hat{g}}_{j}\left(x\right)$ is

(11) $$∥{\hat{g}}_{j}{∥}_{n}^{2}=\frac{1}{n}\sum_{i=1}^{n}{\left({\hat{g}}_{j}\left(x_{i}\right)\right)}^{2}=\frac{1}{n}\sum_{i=1}^{n}{\left({\hat{\alpha }}_{z}^{T}\partial_{j}K_{n}\left(x_{i}\right)\right)}^{2}.$$

Then, the identified active set can be written as

(12) $$\hat{S}=\{j:∥{\hat{g}}_{j}{∥}_{n}^{2}>v_{n}\},$$

where $v_{n}$ is a positive threshold selected under the sample-adaptive tuning framework [37].

## 3. Generalization-Bound and Variable Selection Consistency

This section establishes the theoretical guarantees on generalization ability and variable selection for the proposed RGVS. Firstly, we introduce some necessary assumptions.

**Assumption** **1.** *The representing function ϕ associated with modal kernel $K_{\sigma }:R\times R\to R^{+}$ satisfies: (i) ϕ is bounded with $\int_{R}\phi \left(u\right)du=1$, ϕ(u)=ϕ(−u) and ϕ(u)≤ϕ(0),∀u∈R; (ii)ϕ(·) is differentiable with $∥\phi^{\prime }{∥}_{\infty }<\infty$ and $\int_{R}u^{2}\phi \left(u\right)du<\infty$.*

Observe that some smoothing kernels meet Assumption 1, such as Gaussian kernel and Logistic kernel, etc.

**Assumption** **2.** *The conditional density function $P_{\epsilon |X}$ is second-order differentiable and $∥P_{\epsilon |X}^{\prime \prime }{∥}_{\infty }$ is bounded.*

Assumption 2 has been used in [19,20], which assures upper bound on $|R\left(f\right)-R^{\sigma }\left(f\right)|$ together with Assumption 1.

**Assumption** **3.** Let $C^{s}$ be a space of s-times continuous differentiable functions. Assume that $\underset{x\in X}{\sup}\sqrt{K(x,x)}<\infty$ with $K\in C^{s}$ with s>0, and for a given constant M, the target function satisfies $f^{*}\in H_{K}$ with $∥f^{*}{∥}_{\infty }\leq M$.

Assumption 3 has been used extensively in learning theory literatures, see, e.g., [38,39,40,41,42,43,44]. In particular, the Gaussian kernel belongs to $C^{\infty }$.

Our error analysis begins with the following inequality in [19], where the relationship between $R^{\sigma }\left(f\right)$ and R(f) is provided.

**Lemma** **1.** *Under Assumptions 1–2, there holds*

$$|R\left(f^{*}\right)-R\left(f\right)-\left(R^{\sigma }\left(f^{*}\right)-R^{\sigma }\left(f\right)\right)|\leq c_{1}\sigma^{2}$$

*for any measurable function f: X→R, where $c_{1}=\left|\right|P_{\epsilon |x}^{\prime \prime }{\left|\right|}_{\infty }\int_{R}u^{2}\phi \left(u\right)du$.*

This indicates us to bound the excess risk $R\left(f^{*}\right)-R\left(f\right)$ via estimating $R^{\sigma }\left(f^{*}\right)-R^{\sigma }\left(f_{z}\right)$. To be specific, we further make an error decomposition as follows.

**Lemma** **2.** *Under Assumptions 1–3, there holds*

$$R\left(f^{*}\right)-R\left(f_{z}\right)\leq R^{\sigma }\left(f^{*}\right)-R^{\sigma }\left(f_{z}\right)-\left(R_{z}^{\sigma }\left(f^{*}\right)-R_{z}^{\sigma }\left(f_{z}\right)\right)+\lambda ||f^{*}{\left|\right|}_{K}^{2}+c_{1}\sigma^{2}.$$

**Proof.**  According to the definition of $f_{z}$ in (9), we have

$$R_{z}^{\sigma }\left(f^{*}\right)-\lambda ∥f^{*}{∥}_{K}^{2}-(R_{z}^{\sigma }\left(f_{z}\right)-\lambda ∥f_{z}{∥}_{K}^{2})\leq 0.$$

Then, we can deduce that

$$\begin{array}{ccc} R^{\sigma }\left(f^{*}\right)-R^{\sigma }\left(f_{z}\right) & = & R^{\sigma }\left(f^{*}\right)-R_{z}^{\sigma }\left(f^{*}\right)+R_{z}^{\sigma }\left(f^{*}\right)-\lambda ∥f^{*}{∥}_{K}^{2}+\lambda {∥f^{*}∥}_{K}^{2}\\ & & -(R_{z}^{\sigma }\left(f_{z}\right)-\lambda ∥f_{z}{∥}_{K}^{2})-\lambda ∥f_{z}{∥}_{K}^{2}+R_{z}^{\sigma }\left(f_{z}\right)-R^{\sigma }\left(f_{z}\right)\\ & \leq & R^{\sigma }\left(f^{*}\right)-R_{z}^{\sigma }\left(f^{*}\right)+R_{z}^{\sigma }\left(f_{z}\right)-R^{\sigma }\left(f_{z}\right)\\ & & +\left\{R_{z}^{\sigma }\left(f^{*}\right)-\lambda ∥f^{*}{∥}_{K}^{2}-(R_{z}^{\sigma }\left(f_{z}\right)-\lambda ∥f_{z}{∥}_{K}^{2})\right\}+\lambda {∥f^{*}∥}_{K}^{2}\\ & \leq & R^{\sigma }\left(f^{*}\right)-R^{\sigma }\left(f_{z}\right)-\left(R_{z}^{\sigma }\left(f^{*}\right)-R_{z}^{\sigma }\left(f_{z}\right)\right)+\lambda {∥f^{*}∥}_{K}^{2}. \end{array}$$

This together with Lemma 1 yields the desired result. □

Observe that $R^{\sigma }\left(f^{*}\right)-R^{\sigma }\left(f_{z}\right)-\left(R_{z}^{\sigma }\left(f^{*}\right)-R_{z}^{\sigma }\left(f_{z}\right)\right)$ characterizes the divergence between the data-free risk $R^{\sigma }\left(f\right)$ and the empirical risk $R_{z}^{\sigma }\left(f\right)$. To establish its uniform estimation, we need to give the upper bound of $∥f_{z}{∥}_{K}$ firstly.

According to the definition of $f_{z}$, we have

$$R_{z}^{\sigma }\left(0\right)\leq R_{z}^{\sigma }\left(f_{z}\right)-\lambda {∥f_{z}∥}_{K}^{2}.$$

Then,

$$∥f_{z}{∥}_{K}\leq \sqrt{\frac{R_{z}^{\sigma }\left(f_{z}\right)-R_{z}^{\sigma }\left(0\right)}{\lambda }}\leq \sqrt{\frac{{∥\phi ∥}_{\infty }}{\lambda \sigma }}.$$

**Lemma** **3.** *For $f_{z}$ in (9), there holds*

$$∥f_{z}{∥}_{K}\leq \sqrt{\frac{{∥\phi ∥}_{\infty }}{\lambda \sigma }}.$$

Lemma 3 tells us that $f_{z}\in B_{r}$ with $r=\sqrt{\frac{{∥\phi ∥}_{\infty }}{\lambda \sigma }}$ for any $z\in Z^{n}$, where $B_{r}=\left\{f\in H_{K}:{∥f∥}_{K}\leq r\right\}$. This motivates us to measure the capacity of $B_{r}$ through the empirical covering number [45].

**Definition** **1.** *Suppose that F is a set of functions on $x=\{x_{1},\ldots ,x_{n}\}$ with the $\ell_{2}$-empirical metric $d_{2,x}\left(f,g\right)={\left(\frac{1}{n}\sum_{i=1}^{n}{\left(f\left(x_{i}\right)-g\left(x_{i}\right)\right)}^{2}\right)}^{\frac{1}{2}},\;\forall \;f,g\in F$. Then, the $\ell_{2}$-empirical covering number of function set F is defined as*

$$N_{2}\left(F,\epsilon \right)=\underset{n\in N}{\sup}\underset{x}{\sup}N_{2,x}\left(F,\epsilon \right),\epsilon >0,$$

*where*

$$N_{2,x}\left(F,\epsilon \right)=\inf\left\{l\in N:\exists {\left\{f_{j}\right\}}_{j=1}^{l}\subset F,s.t.,F\subset \cup_{j=1}^{l}B\left(f_{j},\epsilon \right)\right\}$$

*with $B\left(f_{j},\epsilon \right)=\left\{f\in F:d_{2,x}\left(f,f_{j}\right)<\epsilon \right\}$*

Next, we introduce a concentration inequality established in [46].

**Lemma** **4.** *Let T be a function set associated with function t. Suppose that there are some constants $B,c_{s},c_{\theta }>0$ and s∈[0,1] satisfying ${∥t∥}_{\infty }\leq B$, $Et^{2}\leq c_{s}{\left(Et\right)}^{s}$ for any t∈T. If for 0<θ<2 and $\log N_{2}\left(T,\epsilon \right)\leq c_{\theta }\epsilon^{-\theta },\forall \epsilon >0$, then for any 0<δ<1 and given $z={\left\{z_{i}\right\}}_{i=1}^{n}\subset Z$, there holds*

$$\begin{array}{c} Et-\frac{1}{n}\sum_{i=1}^{n}t\left(z_{i}\right)\leq \frac{1}{2}\eta^{1-s}{\left(Et\right)}^{s}+c_{\theta }^{\prime }\eta +2{\left(\frac{c_{s}\log\left(1/\delta \right)}{n}\right)}^{\frac{1}{2-s}}+\frac{18B\log(1/\delta )}{n},\forall t\in T, \end{array}$$

*where $c_{\theta }^{\prime }$ is a constant only depending on θ and*

$$\eta =\max\left\{c_{s}^{\frac{2-\theta }{4-2s+\theta s}}{\left(\frac{c_{\theta }}{n}\right)}^{\frac{2}{4-2s+\theta s}},B^{\frac{2-\theta }{2+\theta }}{\left(\frac{c_{\theta }}{n}\right)}^{\frac{2}{2+\theta }}\right\}.$$

**Theorem** **1.** *Under Assumptions 1–3, taking $\sigma =n^{-\frac{1}{5}}$ and $\lambda =n^{-\frac{2}{5}}$, we have for any 0<δ<1*

$$\begin{array}{c} R\left(f^{*}\right)-R\left(f_{z}\right)\leq Cn^{-\zeta }\log\left(\frac{1}{\delta }\right) \end{array}$$

*with confidence at least 1−δ, where $\zeta =\min\left\{\frac{8-9\theta }{20},\frac{8-6\theta }{5(2+\theta )},\frac{2}{5}\right\}$ and*

(13) $$\begin{array}{c} \theta =\left\{\begin{array}{cc} \frac{2p}{p+2s} & 0<s\leq 1\\ \frac{2p}{p+2} & 1<s\leq 1+\frac{p}{2}\\ \frac{p}{s} & s>1+\frac{p}{2}. \end{array}\right. \end{array}$$

**Proof.**  Denote a function-based random variable set by

$$\begin{array}{c} T=\left\{t\left(z\right):=t_{f}\left(z\right)=\frac{1}{\sigma }\left(\phi \left(\frac{y-f^{*}\left(x\right)}{\sigma }\right)-\phi \left(\frac{y-f(x)}{\sigma }\right)\right):f\in B_{r}\right\}. \end{array}$$

Under Assumption 1, for any $f_{1},f_{2}\in B_{r}$, we have

$$\begin{array}{ccc} |t_{f_{1}}\left(z\right)-t_{f_{2}}\left(z\right)| & = & \frac{1}{\sigma }|\phi \left(\frac{y-f_{1}\left(x\right)}{\sigma }\right)-\phi \left(\frac{y-f_{2}\left(x\right)}{\sigma }\right)|\\ & \leq & \frac{∥\phi^{\prime }{∥}_{\infty }}{\sigma }|\frac{y-f_{1}\left(x\right)}{\sigma }-\frac{y-f_{2}\left(x\right)}{\sigma }|\\ & \leq & \frac{∥\phi^{\prime }{∥}_{\infty }}{\sigma^{2}}\left|f_{1}\left(x\right)-f_{2}\left(x\right)\right|. \end{array}$$

Combining the above inequality and the properties of empirical covering number [40,41], we have

(14) $$\log N_{2}\left(T,\epsilon \right)\leq \log N_{2}\left(B_{1},\frac{\epsilon \sigma^{2}}{r∥\phi^{\prime }{∥}_{\infty }}\right)\leq C_{\theta }^{\prime }r^{\theta }\sigma^{-2\theta }\epsilon^{-\theta },$$

where θ is defined in (13).

According to Assumption 1, there exists ${∥t∥}_{\infty }\leq \frac{{∥\phi ∥}_{\infty }}{\sigma }$. Furthermore, we get

(15) $$\begin{array}{ccc} Et^{2} & = & \frac{{∥\phi ∥}_{\infty }}{\sigma }E\left[\frac{1}{\sigma }\phi \left(\frac{y-f^{*}}{\sigma }\right)-\frac{1}{\sigma }\phi \left(\frac{y-f(x)}{\sigma }\right)\right]=\frac{{∥\phi ∥}_{\infty }}{\sigma }\left(R^{\sigma }\left(f^{*}\right)-R^{\sigma }\left(f\right)\right)\\ & \leq & \frac{{∥\phi ∥}_{\infty }}{\sigma }\left(P_{E_{f^{*}}}\left(0\right)-P_{E_{f}}\left(0\right)+c_{1}\sigma^{2}\right)\leq \frac{{∥\phi ∥}_{\infty }}{\sigma }\left(P_{E_{f^{*}}}\left(0\right)-P_{E_{f}}\left(0\right)\right)+{∥\phi ∥}_{\infty }c_{1}\sigma \;\\ & \leq & \sigma^{-1}c_{2}+\sigma c_{3}, \end{array}$$

where $c_{2}={∥\phi ∥}_{\infty }\left(P_{E_{f^{*}}}\left(0\right)-P_{E_{f}}\left(0\right)\right)$ and $c_{3}=c_{1}{∥\phi ∥}_{\infty }$ are the bounded constants.

Recalling (14) and (15), we know Lemma 4 holds true for t∈T with $c_{\theta }=c_{\theta }^{\prime }r^{\theta }\sigma^{-2\theta }$, $B=\frac{{∥\phi ∥}_{\infty }}{\sigma }$, s=0, and $c_{s}=c_{2}\sigma^{-1}+c_{3}\sigma$. That is to say, for any t∈T and 0<δ<1, with confidence 1−δ

(16) $$\begin{array}{cc} & R^{\sigma }\left(f^{*}\right)-R^{\sigma }\left(f\right)-\left(R_{z}^{\sigma }\left(f^{*}\right)-R_{z}^{\sigma }\left(f\right)\right)\\ \leq & \left(\frac{1}{2}+c_{\theta }^{\prime }\right)\max\left\{{\left(c_{2}\sigma^{-1}+c_{3}\sigma \right)}^{\frac{2-\theta }{4}}{\left(\frac{c_{\theta }^{\prime }r^{\theta }\sigma^{-2\theta }}{n}\right)}^{\frac{1}{2}},{\left(\frac{{∥\phi ∥}_{\infty }}{\sigma }\right)}^{\frac{2-\theta }{2+\theta }}{\left(\frac{c_{\theta }^{\prime }r^{\theta }\sigma^{-2\theta }}{n}\right)}^{\frac{2}{2+\theta }}\right\}\\ & +2\sqrt{\frac{\left(c_{2}\sigma^{-1}+c_{3}\sigma \right)\log\left(1/\delta \right)}{n}}+\frac{{18∥\phi ∥}_{\infty }\log\left(1/\delta \right)}{n\sigma }. \end{array}$$

Combining Lemma 2 and (16) with $r=\sqrt{{∥\phi ∥}_{\infty }/\lambda \sigma }$, we have with confidence at least 1−δ

(17) $$\begin{array}{c} R\left(f^{*}\right)-R\left(f_{z}\right)\leq C_{n,\sigma ,\lambda }\log\left(\frac{1}{\delta }\right)\left(\max\left\{\sigma^{-\frac{2+5\theta }{4}}n^{-\frac{1}{2}},\sigma^{-\frac{2+4\theta }{2+\theta }}n^{-\frac{2}{2+\theta }}\lambda^{-\frac{\theta }{2+\theta }}\right\}+n^{-\frac{1}{2}}\sigma^{-\frac{1}{2}}+\lambda +\sigma^{2}\right), \end{array}$$

where $C_{n,\sigma ,\lambda }$ is positive constants independently of n,σ,λ.

Setting $\sigma^{2}=n^{-\frac{1}{2}}\sigma^{-\frac{1}{2}}$ and $\lambda =\sigma^{2}$, we have $\sigma =n^{-\frac{1}{5}}$ and $\lambda =n^{-\frac{2}{5}}$. Putting these selected parameters into (17), we get the desired estimation. □

Theorem 1 provides the upper bound to the excess risk of $f_{z}$ under the MRC, which extends the previous ERM-based analysis in [19] to the regularized learning scheme. In addition, we can further bound $∥f_{z}-f^{*}{∥}_{L_{\rho_{X}}^{2}}^{2}$ after imposing Assumption 3 in [19].

**Corollary** **1.** *Let the conditions of Theorem 1 be true. Assume that $K\in C^{\infty }$, we have*

$$\begin{array}{c} R\left(f^{*}\right)-R\left(f_{z}\right)\leq O\left(n^{-\frac{2}{5}}\log\left(1/\delta \right)\right) \end{array}$$

*with confidence at least 1−δ.*

The learning rate derived in Corollary 1 is faster than $O(n^{-\frac{1}{7}})$ for the linear regularized modal regression [20]. Meanwhile, it should be noted that some kernel functions meet $K\in C^{\infty }$, e.g., Gaussian kernel, Sigmoid kernel, and Logistic kernel.

Since the proposed RGVS employs the non-convex mode-induced loss, our variable selection analysis is completely different from kernel method with least-squares loss [12]. Here, we introduce the following self-calibration inequality, which addresses that a weak convergence on risk implies a strong convergence in kernel-norm under certain conditions.

**Assumption** **4.** *For any given σ and $B_{r}$ with $r={∥\phi ∥}_{\infty }^{\frac{1}{2}}n^{\frac{1}{4}}\sigma^{-\frac{1}{4}}$, there exists a universal constant $C_{1}>0$ such that*

$$R^{\sigma }\left(f^{*}\right)-R^{\sigma }\left(f\right)\geq C_{1}{∥f^{*}-f∥}_{K}^{2},\forall f\in B_{r}.$$

Assumption 4 characterizes the concentration of our estimator near $f^{*}$ with the kernel-norm metric. Indeed, the current restriction is related to Assumption 4 in [12], Theorem 2.7 in [47] for quartile regression, and the so-called RNI condition in [48,49] as well.

In addition, the following condition is required, which implies that the gradient function associated with truly informative variables is separated well from zero. Similar assumptions can also be found in [12,50]. For simplicity, we denote $∥g_{j}{∥}_{2}:=\underset{X}{\inf}{\left(g_{j}\left(x\right)\right)}^{2}d\rho_{X}\left(x\right)$.

**Assumption** **5.** *There exists some constant $C_{2}>0$ such that*

$$\min_{j\in S^{*}}{∥g_{j}^{*}∥}_{2}^{2}>C_{2}n^{-\min\{\frac{1}{8},\frac{4-\theta }{16+8\theta }\}}.$$

**Theorem** **2.** Let Assumptions 1–5 be true. For any given $\sigma >\max\{n^{-\frac{4-\theta }{8+14\theta }},n^{-\frac{2}{4+10\theta }}\}$, set $\lambda =n^{-\frac{1}{2}}\sigma^{-\frac{1}{2}}$ in (9) and $v_{n}=C_{2}n^{-min\{\frac{1}{8},\frac{4-\theta }{16+8\theta }\}}$ in (12). Then, $Prob\{\hat{S}=S^{*}\}\to 1\;as\;n\to \infty$.

**Proof.**  As shown in [12], by direct computation, there holds

(18) $$|∥{\hat{g}}_{j}{∥}_{n}^{2}-{∥g_{j}^{*}∥}_{2}^{2}|\leq a_{1}\left(3∥f_{z}-f^{*}{∥}_{K}+{∥{\hat{D}}_{j}^{*}{\hat{D}}_{j}-D_{j}^{*}D_{j}∥}_{HS}\right),\forall j,$$

where HS denotes the Hilbert-Schmidt operator on $H_{K}$, $D_{j}^{*}D_{j}f=\int \alpha_{j}K_{x}g_{j}\left(x\right)d\rho_{X}\left(x\right)$, ${\hat{D}}_{j}^{*}{\hat{D}}_{j}f=\frac{1}{n}\sum_{i=1}^{n}\alpha_{j}K_{x_{j}}g_{j}\left(x_{i}\right)$, and $a_{1}$ is a positive constant. The concentration inequality for kernel operator in [17] states that

(19) $$∥\hat{D_{j}^{*}}\hat{D_{j}}-D_{j}^{*}D_{j}{∥}_{HS}⩽\sqrt{\frac{8\kappa^{2}}{n}\log\left(\frac{4p}{\delta }\right)}$$

with confidence 1−δ.

Meanwhile, with the similar proof of Theorem 1, we can deduce that

$$R^{\sigma }\left(f^{*}\right)-R^{\sigma }\left(f_{z}\right)⩽a_{2}\left\{\log\left(1/\delta \right)\max\left\{\sigma^{-\frac{2+5\theta }{4}}n^{-\frac{1}{2}},\sigma^{-\frac{2+4\theta }{2+\theta }}n^{-\frac{2+4\theta }{2+\theta }}\right\}+n^{-\frac{1}{2}}\sigma^{-\frac{1}{2}}+\lambda \right\}$$

with confidence at least 1−δ, where $a_{2}$ is a positive constant. Setting $\lambda =n^{-\frac{1}{2}}\sigma^{-\frac{1}{2}},\sigma^{-\frac{2+5\theta }{4}}n^{-\frac{1}{4}}⩽1$ and $\sigma^{-\frac{4+7\theta }{4+2\theta }}n^{-\frac{4-\theta }{8+4\theta }}⩽1$, we further get

$$R^{\sigma }\left(f^{*}\right)-R^{\sigma }\left(f_{z}\right)⩽a_{3}\log\left(1/\delta \right)n^{-\min\{\frac{1}{4},\frac{4-\theta }{8+4\theta }\}}$$

with confidence 1−δ. This excess risk estimation together with Assumption 4 implies that

(20) $$∥f_{z}-f^{*}{∥}_{K}^{2}⩽a_{3}C_{1}^{-1}\log\left(1/\delta \right)n^{-\min\{\frac{1}{4},\frac{4-\theta }{8+4\theta }\}}$$

with confidence 1−δ, where $a_{3}$ is a positive constant.

Combining (18)–(20), we have with confidence 1−δ

(21) $$\forall j,|∥\hat{g_{j}}{∥}_{n}^{2}-{∥g_{j}^{*}∥}_{2}^{2}|⩽a_{4}\sqrt{log(p/\delta )}n^{-\min\{\frac{1}{8},\frac{4-\theta }{6+8\theta }\}},$$

where $a_{4}>0$ is a constant independently of n,δ,λ.

Now we turn to investigate the relationship between $\hat{S}$ in (12) and $S^{*}$ in (2). Firstly, we suppose there exists some $j^{\prime }\in S^{*}$ but $j^{\prime }\notin \hat{S}$. That is to say $∥{\hat{g}}_{j^{\prime }}{∥}_{n}^{2}⩽v_{n}$. By Assumption 5 with $C_{2}=2a_{4}\sqrt{\log(p/\delta )}$, we have

$$|∥{\hat{g}}_{j^{\prime }}{∥}_{n}^{2}-∥g_{j^{\prime }}^{*}{∥}_{2}^{2}|\geq ∥g_{j^{\prime }}^{*}{∥}^{2}-{∥{\hat{g}}_{j^{\prime }}∥}_{n}^{2}>a_{4}\sqrt{\log(p/\delta )}n^{-\min\{\frac{1}{8},\frac{4-\theta }{16+8\theta }\}},$$

which contradicts with (21). This implies that $S^{*}\subset \hat{S}$ with confidence 1−δ.

Secondly, we suppose there exists some $j^{\prime }\in \hat{S}$ but $j^{\prime }\notin S^{*}$. This means $∥g_{j^{\prime }}^{*}{∥}_{2}^{2}=0$ and $∥{\hat{g}}_{j^{\prime }}{∥}_{n}^{2}>v_{n}$. Then

$$|∥{\hat{g}}_{j^{\prime }}{∥}_{n}^{2}-∥g_{j^{\prime }}^{*}{∥}_{2}^{2}|={∥{\hat{g}}_{j^{\prime }}∥}_{n}^{2}>v_{n}=a_{4}\sqrt{\log(p/\delta )}n^{-\min\{\frac{1}{8},\frac{4-\theta }{16+8\theta }\}},$$

which contradicts with (21) with confidence 1−δ. Therefore, the desired property follows by combining these two results. □

Theorem 2 demonstrates that the identified variables are consistent with truly informative variables with probability 1 as n→∞. This result guarantees the variable selection performance of our approach, provided that the active variables have enough gradient signal. In the future, it is necessary to further investigate the self-calibration assumption for RMR in RKHS.

When choosing Gaussian kernel as the modal kernel, the modal regression is consistent with regression under the maximum correntropy criterion (MCC) [36]. In terms of the breakdown point theory, Theorem 24 in [19] established the robustness characterization of kernel regression under MCC and Theorem 3 in [36] provided robust analysis for RMR. These results imply the robustness of our approach.

## 4. Optimization Algorithm

With the help of half-quadratic (HQ) optimization [32], the maximization problem (9) can be transformed into a weighted least-squares problem, and then get the estimator via the ADMM [18]. Indeed, the kernel-based RMR (9) can be implemented directly by the optimization strategy in [36,51] for Gaussian kernel-based modal representation, and in [20] for Epanechnikov kernel-based modal representation. For completeness, we provide the optimization steps of (9) associated with Logistic kernel-based density estimation.

Consider a convex function

$$f\left(a\right)=1/\left(\exp\left(\sqrt{a}\right)+2+\exp\left(-\sqrt{a}\right)\right),\;a>0.$$

As illustrated in [52], a convex function f(a) and its convex conjugate function g(b) satisfy

(22) $$\begin{array}{c} f\left(a\right)=\max_{b}\left(ab-g\left(b\right)\right). \end{array}$$

According to the Logistic-based representation ϕ and (22), we have

(23) $$\phi \left(t\right)=f\left(t^{2}\right)=\max_{b}\left(t^{2}b-g\left(b\right)\right),t\in R.$$

Applying (23) into (10), we can obtain the augmented objective function

(24) $$\max_{\alpha \in R^{n},b\in R^{n}}\left\{\frac{1}{n\sigma }\sum_{i=1}^{n}\left(b_{i}{\left(\frac{y_{i}-\alpha^{T}K_{n}\left(x_{i}\right)}{\sigma }\right)}^{2}-g\left(b_{i}\right)\right)-\lambda \alpha^{T}K\alpha \right\},$$

where $\alpha ={\left(\alpha_{1},\ldots ,\alpha_{n}\right)}^{T}\in R^{n}$, and $b={\left(b_{1},\ldots ,b_{n}\right)}^{T}\in R^{n}$ is the auxiliary vector. Then the maximization problem (24) can be solved by the following iterative optimization algorithm.

According to Theorem 1 in [20], we have $\arg\max_{b}\left(ab-g\left(b\right)\right)=f^{\prime }\left(a\right)$. Then, for a fixed α, $b_{i}$ can be updated by $b_{i}=f^{\prime }\left({\left(\frac{y_{i}-\alpha^{T}K_{n}\left(x_{i}\right)}{\sigma }\right)}^{2}\right)$. While *b* is settled down, update α via

(25) $$\arg\max_{\alpha \in R^{n}}\left\{\sum_{i=1}^{n}\frac{b_{i}}{\sigma }{\left(y_{i}-\alpha^{T}K_{n}\left(x_{i}\right)\right)}^{2}-\lambda \alpha^{T}K\alpha \right\}.$$

For $K={\left(K\left(x_{i},x_{j}\right)\right)}_{i,j=1}^{n}\in R^{n\times n}$ and $Y=\left(y_{1},\ldots ,y_{n}\right)\in R^{n}$, the problem (25) can be rewritten as

$$\arg\min_{\alpha \in R^{n}}{\left(Y-K\alpha \right)}^{T}diag\left(-\frac{b}{\sigma }\right)\left(Y-K\alpha \right)+\lambda \alpha^{T}K\alpha$$

where diag(·) is an operator that transforms the vector into a diagonal matrix. By setting $\partial [{\left(Y-K\alpha \right)}^{T}diag\left(-b/\sigma \right)\left(Y-K\alpha \right)+\lambda \alpha^{T}K\alpha ]/\partial \alpha =0$, we have

(26) $$\alpha =4{\left(Kdiag\left(-\frac{b}{\sigma }\right)+\lambda I\right)}^{-1}diag\left(-\frac{b}{\sigma }\right)Y.$$

When α is obtained from (26), we can calculate the gradient-based measure $∥{\hat{g}}_{j}{∥}_{n}^{2}$ by (11) directly. Then we apply a pre-specified threshold $v_{n}$ to identify the truly active set ${\hat{S}}_{n}=\{j:∥{\hat{g}}_{j}{∥}_{n}^{2}>v_{n}\}$. Here, the threshold $v_{n}$ is selected by the stability-based criterion [37], which include two steps as below. Firstly, the training samples are randomly divided into two subsets, and the identified active variable sets $J_{z,1k}$ and $J_{z,2k}$ are obtained under given $v_{n}$ for the *k*-th splitting of training samples. Then, the threshold $v_{n}$ is updated by maximizing the Cohen kappa statistical measure $\frac{1}{T}\sum_{k=1}^{T}\kappa \left(J_{z,1k},J_{z,2k}\right)$.

The optimization steps of RGVS are summarized in Algorithm 1.

**Algorithm 1**: Optimization algorithm of RGVS with Logistic kernel**Input**: Samples z, the modal representation ϕ (Logistic kernel), Mercer kernel *K*;**Initialization**: t=0, α, bandwidth σ, Max-iter $=10^{2}$, $\varepsilon =10^{-3}$;**Obtain $f_{z}$ in RKHS**:      **While**
α not converged and t< Max-iter;            1. Fixed $\alpha^{t}$, update $b_{i}^{t+1}=-\frac{\exp(q)-\exp(-q)}{2q{\left(\exp\left(q\right)+2+\exp\left(-q\right)\right)}^{2}},q=\frac{y_{i}-K_{n}^{T}\left(x_{i}\right)\alpha^{t}}{\sigma }$;            2. Fixed $b^{t+1}$, update $\alpha^{t+1}=4{\left(Kdiag\left(-\frac{b^{t+1}}{\sigma }\right)+\lambda I\right)}^{-1}diag\left(-\frac{b^{t+1}}{\sigma }\right)Y$;            3. Check the convergence condition: $∥\alpha^{t+1}-\alpha^{t}{∥}^{2}<\varepsilon$;            4. t←t+1;      **End While**      **Output**: ${\hat{\alpha }}_{z}=\alpha^{t+1}$;**Variable Selection**: ${\hat{S}}_{n}=\{j:\frac{1}{n}\sum_{i=1}^{n}{\left({\hat{\alpha }}_{z}^{T}\partial_{j}K_{n}\left(x_{i}\right)\right)}^{2}>v_{n}$}.**Output**: ${\hat{S}}_{n}$

## 5. Empirical Assessments

This section assesses the empirical performance of our proposed method on simulated and real-world datasets. Three variable selection methods are introduced as the baselines, which include *Least Absolute Shrinkage and Selection Operator* (Lasso) [1], *Sparse Additive Models* (SpAM) [9], and *General Variable Selection Method* (GM) [12].

In all experiments, the RKHS $H_{K}$ associated with Gaussian kernel $K_{h}\left(u,v\right)=\exp\left(-\frac{{∥u-v∥}_{2}^{2}}{2h^{2}}\right)$ is employed as the hypothesis function space. For simplicity, we denote $RGVS_{Gau}$ and $RGVS_{Log}$ as the proposed RGVS method with Gaussian modal kernel and Logistic modal kernel, respectively. In the simulated experiments, we generate three datasets (with identical sample size) independently as the training set, the validation set, and the testing set, respectively. The hyper-parameters are tuned via grid research on validation set, and the corresponding grids are displayed as follows: (i) the regularization parameter λ: $\left\{10^{-3},5\times 10^{-3},10^{-2},5\times 10^{-2},\ldots ,1,5,10\right\}$; (ii) the bandwidth σ and *h*: $\left\{1+10^{-1}i,i=0,1,\ldots ,100\right\}$; (iii) the threshold $v_{n}$: $\{10^{-3+0.1t},t=0,\ldots ,60$}.

### 5.1. Simulated Data

Now we evaluate our approach on two synthetic data used in [12,13]. The first example is a simple additive function and the second one is a function that includes interaction terms.

**Example** **1.** We generate the p-dimension input $x_{i}=\left(x_{i1},\ldots ,x_{ip}\right)$ by $x_{ij}=\frac{W_{ij}+\eta V_{i}}{1+\eta }$, where both $W_{ij}$ and $V_{i}$ are extracted from the uniform distribution U(−0.5,0.5) and η=0.2. The output $y_{i}$ is generated by $y_{i}=f^{*}\left(x_{i}\right)+\epsilon_{i}$, where $f^{*}\left(x_{i}\right)=5x_{i1}+4{\left(x_{i2}-1\right)}^{2}+0.5\sin\left(\pi x_{i3}\right)+\cos\left(\pi x_{i3}\right)+1.5{\left(\sin\left(\pi x_{i3}\right)\right)}^{2}+2.5{\left(\sin\left(\pi x_{i3}\right)\right)}^{3}+2{\left(\cos\left(\pi x_{i3}\right)\right)}^{3}+6\sin\left(\pi x_{i4}\right)/\left(2-\sin\left(\pi x_{i4}\right)\right)$ and $\epsilon_{i}$ is a random noise. Here, we consider the Gaussian noise N(0,1), the Chi-square noise $X^{2}\left(2\right)$, the Student noise t(2), and the Exponential noise E(2), respectively.

**Example** **2.** This example follows the way of Example 1 to generate data. The differences are that $W_{ij}$ and $V_{i}$ are extracted from the same distribution U(0,1) and the true function $f^{*}\left(x_{i}\right)=20x_{i1}x_{i2}x_{i3}+5x_{i4}^{2}+5x_{i5}$.

**Figure 1:**
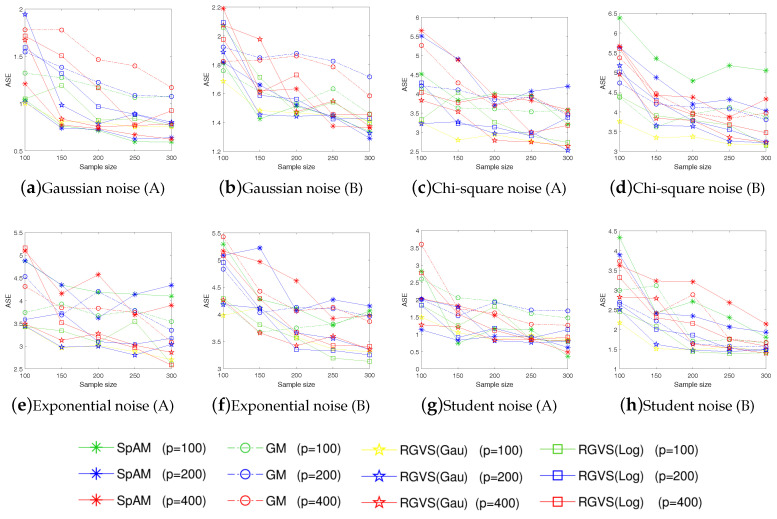
The average squares error (ASE) vs. the sample size *n* under different noise (A and B represent *Example 1*. and *Example 2* respectively).

**Figure 2:**
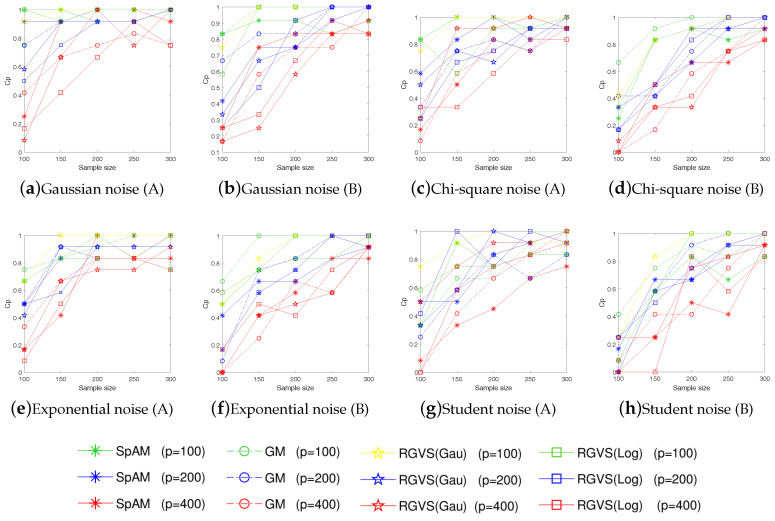
The correct-fitting probability (Cp) vs. the sample size *n* under different noise (A and B represent *Example 1*. and *Example 2* respectively).

For each evaluation, we consider training set with different size n=100,150,200 and dimension p=150. To make sure the results are reliable, each evaluation is repeated 50 times. Since the truly informative variables are usually unknown in practice, we evaluate the algorithmic performance according to the *average squares error*(ASE) defined as $ASE:=\frac{1}{n}\sum_{i=1}^{n}{\left(f^{*}\left(x_{i}\right)-f_{z}\left(x_{i}\right)\right)}^{2}$. To better evaluate the algorithmic performance, we also adopt some metrics used in [12,13] to measure the quality of model regression, e.g., Cp (correct-fitting), SIZE (the average number of selected variables), TP (the average number of the selected true informative variables), FP (the average number of the selected uninformative variables), Up (under-fitting probability), Op (over-fitting probability). The detail result is summarized in Table 2 and Table 3. To further support the competitive performance of the proposed method, we also provide the experimental results on ASE in Figure 1 and Cp in Figure 2 with n=[100:50:300] and p=100,200,400. Figure 1 and Figure 2 show that our method has always performed well with different *n*.

Empirical evaluations on simulated examples verify the promising performance of RGVS on variable selection and regression estimation, even for data with non-Gaussian noises (e.g., the Chi-square noise $X^{2}\left(2\right)$, the Student noise t(2), and the Exponential noise E(2)). Meanwhile, GM and RGVS have similar performance under the Gaussian noise setting, which is consistent with our motivation for algorithmic design.

### 5.2. Real-World Data

We now evaluate our RGVS on Auto-Mpg and Requirements of buildings, which are all collected from UCI. Since the variable number is very limited for the current datasets, 100 irrelative variables are added, which are generated from the distribution of U(−0.5,0.5).

Auto-Mpg data describes the mile per gallon of automobile (MPG). It contains 398 samples and 7 variables, including Cylinders, Displacement, Horsepower, Weight, Acceleration, Model year, and Origin. The second real data sets is obtained to assess the heating load and cooling load requirements of buildings which contains 768 samples and 8 input variables, including Relative Compactness, Surface Area, Wall Area, Roof Area, Overall Height, Orientation, Glazing Area, and Glazing Area Distribution. In particular, it has two response variables (heating load and cooling load).

Now, we use the 5-fold cross validation to tune the hyper-parameters and employ the *relative sum of the squared errors* (RSSE) to measure learning performance. Here $RSSE=\sum_{x\in X_{test}}{\left(f\left(x\right)-f_{z}\left(x\right)\right)}^{2}/\sum_{x\in X_{test}}{\left(f\left(x\right)-E\left(f\right)\right)}^{2}$, where $f_{z}$ is the estimator of *f* and E(f) denotes the average value of *f* on the test set $X_{test}$. Experimental results are reported in Table 4 and Table 5.

As shown in Table 4, our method identifies similar variables as GM, but can achieve the smaller RSSE. At same time, SpAM and Lasso tend to select less variables than GM and RGVS, which may discard the truly informative variable for regression estimation. Table 5 shows RGVS has better performance for both the *Heating Load* data and the *Cooling Load* data. All these empirical evaluations validate the effectiveness of our learning strategy consistently.

## 6. Conclusions

This paper proposes a new RGVS method rooted in kernel modal regression. The main advantages of RGVS are its flexibility on mimicking the decision function and adaptivity on screening the truly active variables. The proposed approach is evaluated by the theoretical analysis on the generalization error and variable selection, and by the empirical results on data experiments. In theory, our method can achieve the polynomial decay rate with $O(n^{-\frac{2}{5}})$. In applications, our model has shown the competitive performance for data with non-Gaussian noises.

## Figures and Tables

**Table 1:** Properties of different regression algorithms.

	Lasso [1]	RMR [20]	SpAM [9]	COSSO [10]	GM [12]	Ours
Learning criterion	MSE	MRC	MSE	MSE	MSE	MRC
Model assumption	linear	linear	additive	additive	model-free	model-free

**Table 2:** The averaged performance on simulated data in *Example 1* (left) and *Example 2* (right).

Noise	(n,p)	Method	SIZE	TP	FP	Up	Op	Cp	ASE	SIZE	TP	FP	Up	Op	Cp	ASE
	(100, 150)	Lasso	3.92	3.92	0.00	0.36	0.00	0.64	1.369	4.40	4.28	0.12	0.44	0.12	0.44	5.112
	n<p	SpAM	4.12	3.92	0.20	0.08	0.16	0.76	1.075	5.02	4.98	0.04	0.04	0.04	**0.92**	1.611
		GM	4.12	3.88	0.24	0.12	0.16	0.72	1.123	5.14	4.98	0.16	0.04	0.12	0.84	1.775
		$RGVS_{Gau}$	4.00	3.92	0.08	0.08	0.04	**0.88**	**1.003**	5.12	5.00	0.12	0.00	0.08	**0.92**	**1.565**
		$RGVS_{Log}$	3.84	3.80	0.04	0.20	0.04	0.76	1.131	5.12	4.92	0.20	0.08	0.16	0.76	1.914
N(0,1)	(150, 150)	Lasso	4.20	3.92	0.04	0.16	0.12	0.72	1.245	4.48	4.28	0.20	0.40	0.16	0.44	4.794
Gaussian Noise	n=p	SpAM	4.00	4.00	0.00	0.00	0.00	**1.00**	**0.804**	5.00	5.00	0.00	0.00	0.00	**1.00**	1.612
		GM	3.96	3.92	0.04	0.08	0.04	0.88	1.011	5.04	5.00	0.04	0.00	0.04	0.96	1.627
		$RGVS_{Gau}$	3.96	3.96	0.00	0.04	0.00	0.96	0.899	5.00	5.00	0.00	0.00	0.00	**1.00**	**1.500**
		$RGVS_{Log}$	3.96	3.80	0.04	0.08	0.04	0.88	1.083	5.02	5.00	0.02	0.00	0.03	0.97	1.622
	(200, 150)	Lasso	4.00	3.92	0.08	0.20	0.00	0.80	1.252	4.52	4.52	0.00	0.40	0.00	0.60	2.507
	n>p	SpAM	4.00	4.00	0.00	0.00	0.00	**1.00**	0.829	5.04	5.00	0.04	0.00	0.02	0.98	1.497
		GM	3.96	3.96	0.00	0.04	0.00	0.96	1.012	5.06	5.00	0.06	0.00	0.04	0.96	1.528
		$RGVS_{Gau}$	4.00	4.00	0.00	0.00	0.00	**1.00**	**0.813**	5.00	5.00	0.00	0.00	0.00	**1.00**	1.485
		$RGVS_{Log}$	3.96	3.96	0.00	0.04	0.00	0.96	0.915	5.00	5.00	0.00	0.00	0.00	**1.00**	**1.453**
	(100, 150)	Lasso	3.72	3.64	0.08	0.36	0.04	0.60	5.854	4.32	3.72	0.60	0.68	0.12	0.20	6.888
	n<p	SpAM	4.24	3.76	0.48	0.24	0.28	0.48	4.342	5.52	5.00	0.52	0.00	0.40	0.60	4.328
		GM	4.24	3.80	0.44	0.20	0.36	0.44	4.012	4.48	4.44	0.04	0.30	0.05	0.65	3.611
		$RGVS_{Gau}$	4.16	3.96	0.20	0.04	0.20	**0.76**	2.937	5.08	4.92	0.16	0.06	0.16	**0.78**	**3.486**
		$RGVS_{Log}$	4.18	3.90	0.28	0.16	0.12	0.72	**2.681**	5.08	4.84	0.24	0.08	0.18	0.74	3.968
$X^{2}\left(2\right)$	(150, 150)	Lasso	5.32	3.84	1.48	0.16	0.48	0.36	5.392	5.16	4.16	1.00	0.60	0.16	0.24	4.503
Chi-square Noise	n=p	SpAM	4.04	3.96	0.08	0.04	0.08	**0.88**	2.765	5.32	5.00	0.32	0.00	0.24	0.76	3.748
		GM	4.00	3.88	0.12	0.12	0.08	0.80	2.873	4.98	4.92	0.06	0.05	0.05	0.90	4.173
		$RGVS_{Gau}$	3.96	3.92	0.04	0.08	0.04	**0.88**	2.809	5.02	5.00	0.02	0.00	0.02	**0.98**	**2.929**
		$RGVS_{Log}$	4.08	3.96	0.12	0.04	0.12	0.84	**2.097**	5.04	5.00	0.04	0.00	0.04	0.96	3.519
	(200, 150)	Lasso	4.24	4.00	0.24	0.00	0.28	0.72	5.805	4.36	4.32	0.04	0.52	0.04	0.44	3.754
	n>p	SpAM	4.08	4.00	0.08	0.00	0.08	0.92	2.463	5.04	5.00	0.04	0.00	0.04	0.96	3.634
		GM	4.04	4.00	0.04	0.00	0.04	**0.96**	2.523	5.18	5.00	0.18	0.00	0.20	0.80	3.816
		$RGVS_{Gau}$	3.96	3.96	0.00	0.04	0.00	**0.96**	2.449	5.00	5.00	0.00	0.00	0.00	**1.00**	**2.989**
		$RGVS_{Log}$	3.96	3.96	0.00	0.04	0.00	**0.96**	**1.738**	5.00	5.00	0.00	0.00	0.00	**1.00**	3.457
	(100, 150)	Lasso	3.46	3.46	0.00	0.60	0.00	0.40	4.631	4.64	4.00	0.64	0.60	0.20	0.20	4.567
	n<p	SpAM	4.28	3.88	0.40	0.12	0.28	0.60	4.599	5.84	5.00	0.84	0.00	0.44	0.56	4.224
		GM	4.20	3.64	0.56	0.36	0.36	0.28	3.941	5.36	4.68	0.68	0.32	0.28	0.40	4.528
		$RGVS_{Gau}$	4.20	3.88	0.32	0.12	0.20	**0.68**	3.274	5.06	4.82	0.24	0.14	0.14	0.72	3.907
		$RGVS_{Log}$	3.96	3.80	0.16	0.20	0.16	0.64	**2.775**	5.12	4.92	0.20	0.04	0.16	**0.80**	**3.667**
E(2)	(150, 150)	Lasso	5.30	3.66	1.64	0.20	0.44	0.36	4.747	5.64	4.16	1.48	0.48	0.28	0.24	4.786
Exponential Noise	n=p	SpAM	4.04	3.96	0.08	0.04	0.08	0.88	3.403	5.28	5.00	0.28	0.16	0.00	0.84	4.969
		GM	4.08	3.96	0.12	0.04	0.12	0.84	3.177	4.98	4.92	0.06	0.08	0.04	0.88	4.129
		$RGVS_{Gau}$	4.00	4.00	0.00	0.00	0.00	**1.00**	2.724	5.02	4.98	0.04	0.02	0.04	**0.94**	**2.964**
		$RGVS_{Log}$	4.00	4.00	0.00	0.00	0.00	**1.00**	**2.643**	5.00	4.96	0.04	0.04	0.04	0.92	3.918
	(200, 150)	Lasso	3.80	3.80	0.00	0.20	0.00	0.80	4.291	4.68	4.60	0.08	0.28	0.08	0.64	3.669
	n>p	SpAM	4.00	4.00	0.00	0.00	0.00	**1.00**	2.988	5.24	5.00	0.24	0.00	0.20	0.80	4.808
		GM	3.96	3.96	0.00	0.04	0.00	0.96	3.016	4.98	4.98	0.00	0.04	0.00	0.96	3.878
		$RGVS_{Gau}$	4.00	4.00	0.00	0.00	0.00	**1.00**	2.884	5.00	5.00	0.00	0.00	0.00	**1.00**	**3.041**
		$RGVS_{Log}$	3.96	3.92	0.04	0.09	0.00	0.91	3.113	4.96	4.96	0.00	0.04	0.00	0.96	3.771
	(100, 150)	Lasso	4.92	3.80	1.12	0.28	0.32	0.40	2.301	6.52	3.92	2.60	0.64	0.20	0.16	6.971
	n<p	SpAM	4.90	3.80	1.1	0.24	0.20	0.56	1.698	7.92	4.72	3.20	0.24	0.44	0.32	4.658
		GM	5.00	3.64	1.36	0.32	0.32	0.36	1.551	5.68	4.32	1.32	0.40	0.32	0.28	3.561
		$RGVS_{Gau}$	4.14	3.94	0.20	0.05	0.10	**0.85**	**0.822**	5.00	4.84	0.16	0.08	0.16	**0.76**	**2.308**
		$RGVS_{Log}$	4.14	3.88	0.26	0.12	0.16	0.72	1.208	4.96	4.80	0.16	0.16	0.12	0.72	2.339
t(2)	(150, 150)	Lasso	5.08	3.72	1.36	0.24	0.40	0.36	1.793	6.32	3.80	2.52	0.68	0.20	0.12	6.020
Student Noise	n=p	SpAM	4.30	4.00	0.30	0.00	0.32	0.68	0.955	5.44	5.00	0.44	0.00	0.28	0.72	2.739
		GM	4.04	3.80	0.24	0.16	0.16	0.68	1.046	5.56	4.60	0.96	0.28	0.08	0.64	2.557
		$RGVS_{Gau}$	4.00	4.00	0.00	0.00	0.00	**1.00**	**0.757**	4.98	4.98	0.00	0.08	0.00	0.92	**1.716**
		$RGVS_{Log}$	3.92	3.88	0.04	0.12	0.04	0.84	1.169	4.96	4.96	0.00	0.04	0.00	**0.96**	1.723
	(200, 150)	Lasso	5.00	3.92	1.08	0.32	0.20	0.48	1.262	5.44	4.36	1.08	0.44	0.28	0.28	2.976
	n>p	SpAM	4.10	4.00	0.10	0.00	0.27	0.73	1.060	5.64	5.00	0.64	0.00	0.28	0.72	2.427
		GM	4.00	3.96	0.04	0.04	0.04	0.92	1.011	5.20	4.72	0.48	0.20	0.04	0.76	2.350
		$RGVS_{Gau}$	4.04	4.00	0.04	0.00	0.10	0.90	**0.681**	5.00	5.00	0.00	0.00	0.00	**1.00**	**1.517**
		$RGVS_{Log}$	4.04	4.00	0.04	0.00	0.04	**0.96**	0.884	4.96	4.96	0.00	0.04	0.00	0.96	1.672

**Table 3:** The averaged performance with simulated data in *Example 1*.

Noise	(n,p)	Method	SIZE	TP	FP	Up	Op	Cp	ASE
	(300, 500)	Lasso	1.98	1.98	0.00	1.00	0.00	0.00	1.98
	n<p	GM	4.04	4.00	0.04	0.00	0.04	**0.96**	0.80
		$RGVS_{Gau}$	4.06	4.00	0.06	0.00	0.06	0.94	**0.63**
		$RGVS_{Log}$	4.14	3.98	0.16	0.01	0.03	**0.96**	0.88
N(0,1)	(500, 500)	Lasso	1.92	1.92	0.00	1.00	0.00	0.00	1.35
Gaussian Noise	n=p	GM	4.06	4.00	0.06	0.00	0.06	0.94	0.78
		$RGVS_{Gau}$	4.02	4.00	0.02	0.00	0.02	**0.98**	**0.59**
		$RGVS_{Log}$	4.04	4.00	0.04	0.00	0.02	**0.98**	0.74
	(700, 500)	Lasso	1.88	1.88	0.00	1.00	0.00	0.00	1.55
	n>p	GM	4.04	4.00	0.04	0.00	0.04	0.96	0.77
		$RGVS_{Gau}$	4.02	4.00	0.02	0.00	0.02	0.98	**0.62**
		$RGVS_{Log}$	4.00	4.00	0.00	0.00	0.00	**1.00**	0.73
	(300, 500)	Lasso	1.80	1.80	0.00	1.00	0.00	0.00	4.45
	n<p	GM	4.18	4.00	0.18	0.00	0.14	0.86	2.92
		$RGVS_{Gau}$	4.09	4.00	0.09	0.00	0.11	**0.89**	2.39
		$RGVS_{Log}$	4.06	3.88	0.18	0.12	0.14	0.74	**1.95**
$X^{2}\left(2\right)$	(500, 500)	Lasso	1.74	1.74	0.00	1.00	0.00	0.00	4.62
Chi-square Noise	n=p	GM	4.14	4.00	0.14	0.00	0.14	0.86	3.01
		$RGVS_{Gau}$	4.08	4.00	0.08	0.00	0.06	**0.94**	2.22
		$RGVS_{Log}$	4.04	3.98	0.06	0.02	0.06	0.92	**1.82**
	(700, 500)	Lasso	1.86	1.86	0.00	1.00	0.00	0.00	4.37
	n>p	GM	4.28	4.00	0.28	0.00	0.24	0.76	2.96
		$RGVS_{Gau}$	4.02	4.00	0.02	0.00	0.02	**0.98**	2.13
		$RGVS_{Log}$	4.02	4.00	0.02	0.00	0.02	**0.98**	**1.72**
	(300, 500)	Lasso	2.04	2.04	0.00	1.00	0.00	0.00	4.25
	n<p	GM	3.94	3.87	0.07	0.13	0.05	0.82	3.14
		$RGVS_{Gau}$	4.02	4.00	0.02	0.00	0.02	**0.98**	2.36
		$RGVS_{Log}$	3.98	3.94	0.04	0.06	0.02	0.92	**1.92**
E(2)	(500, 500)	Lasso	1.94	1.94	0.00	1.00	0.00	0.00	4.34
Exponential Noise	n=p	GM	4.12	4.00	0.12	0.00	0.10	0.90	2.35
		$RGVS_{Gau}$	3.99	3.96	0.03	0.04	0.03	0.93	2.37
		$RGVS_{Log}$	4.02	4.00	0.02	0.00	0.02	**0.98**	**1.71**
	(700, 500)	Lasso	1.90	1.90	0.00	1.00	0.00	0.00	4.67
	n>p	GM	4.08	4.00	0.08	0.00	0.06	0.94	2.33
		$RGVS_{Gau}$	3.99	3.99	0.00	0.01	0.00	**0.99**	**1.74**
		$RGVS_{Log}$	4.05	4.00	0.05	0.00	0.05	0.95	1.92
	(300, 500)	Lasso	1.96	1.96	0.00	1.00	0.00	0.00	4.63
	n<p	GM	3.50	3.46	0.04	0.24	0.04	0.72	2.48
		$RGVS_{Gau}$	4.14	3.94	0.20	0.06	0.10	0.84	**0.82**
		$RGVS_{Log}$	4.00	3.98	0.02	0.02	0.00	**0.98**	0.90
t(2)	(500, 500)	Lasso	1.76	1.76	0.00	0.98	0.00	0.02	3.83
Student Noise	n=p	GM	4.30	4.00	0.30	0.00	0.16	0.84	1.96
		$RGVS_{Gau}$	4.00	4.00	0.00	0.00	0.00	**1.00**	0.76
		$RGVS_{Log}$	4.02	4.00	0.02	0.00	0.01	0.99	**0.75**
	(700, 500)	Lasso	1.96	1.96	0.00	0.96	0.00	0.04	2.46
	n>p	GM	4.06	4.00	0.06	0.00	0.04	0.96	1.95
		$RGVS_{Gau}$	4.04	4.00	0.04	0.00	0.06	0.94	**0.68**
		$RGVS_{Log}$	4.00	4.00	0.00	0.00	0.00	**1.00**	0.74

**Table 4:** Learning performance on Auto-Mpg.

Variable	CyL	DISP	HPOWER	WEIG	ACCELER	YEAR	ORIGN	RSSE(std)
Lasso	-	-	-	✔	-	✔	-	0.5918(0.3762)
SpAM	✔	✔	-	✔	-	-	-	0.2754(0.0191)
GM	✔	✔	✔	✔	-	✔	✔	0.2547(0.0313)
RGVS${}_{Gau}$	✔	✔	✔	✔	-	✔	-	0.1425(0.0277)
RGVS${}_{Log}$	✔	✔	✔	✔	-	✔	✔	**0.1379(0.0183)**

**Table 5:** Learning performance on Heating Load (UP) and Cooling Load (DOWN).

Variable	RC	SA	WA	RA	OH	ORIENT	GA	GAD	RSSE(std)
Lasso	-	-	-	-	✔	-	✔	-	0.1739(0.0801)
SpAM	-	-	-	✔	✔	-	-	-	0.1684(**0.0045**)
GM	✔	✔	✔	✔	✔	-	-	-	0.1244(0.0383)
RGVS${}_{Gau}$	-	✔	✔	✔	✔	-	✔	-	**0.0935**(0.0099)
RGVS${}_{Log}$	-	-	✔	✔	✔	-	✔	-	0.1110(0.0066)
Lasso	-	-	✔	-	✔	-	✔	-	0.2119(0.0926)
SpAM	-	-	-	✔	✔	-	-	-	0.1910(0.0131)
GM	✔	✔	✔	✔	✔	-	-	-	0.1515(0.0120)
RGVS${}_{Gau}$	-	✔	✔	✔	✔	-	✔	-	**0.1339**(0.0116)
RGVS${}_{Log}$	✔	✔	✔	✔	✔	-	-	-	0.1368(**0.0077**)
